# Highly efficient, perfect, large angular and ultrawideband solar energy absorber for UV to MIR range

**DOI:** 10.1038/s41598-022-22951-1

**Published:** 2022-10-27

**Authors:** Shobhit K. Patel, Arun Kumar Udayakumar, G. Mahendran, B. Vasudevan, Jaymit Surve, Juveriya Parmar

**Affiliations:** 1 Department of Computer Engineering, Marwadi University, Rajkot, Gujarat 360003 India; 2 Department of EEE, SRM Institute of Science and Technology, Ramapuram Campus, Chennai, Tamilnadu 600089 India; 3 Department of EEE, Kathir College of Engineering, Coimbatore, Tamilnadu 641062 India; 4 Department of Electronics and Communication Engineering, St. Joseph’s College of Engineering, OMR, Chennai, 600119 India; 5 Department of Electrical Engineering, Marwadi University, Rajkot, Gujarat 360003 India; 6 Department of Electronics and Communication Engineering, Marwadi University, Rajkot, Gujarat 360003 India; 7 Department of Mechanical and Materials Engineering, University of Nebraska-Lincoln, 1400 R St., Nebraska, 68588 USA

**Keywords:** Solar energy, Solar thermal energy, Metamaterials, Computational methods

## Abstract

Although different materials and designs have been tried in search of the ideal as well as ultra-wideband light absorber, achieving ultra-broadband and robust unpolarized light absorption over a wide angular range has proven to be a major issue. Light-field regulation capabilities provided by optical metamaterials are a potential new technique for perfect absorbers. It is our goal to design and demonstrate an ultra-wideband solar absorber for the ultraviolet to a mid-infrared region that has an absorptivity of TE/TM light of 96.2% on average. In the visible, NIR, and MIR bands of the solar spectrum, the absorbed energy is determined to be over 97.9%, above 96.1%, and over 95%, respectively under solar radiation according to the Air Mass Index 1.5 (AM1.5) spectrum investigation. In order to achieve this wideband absorption, the TiN material ground layer is followed by the SiO_2_ layer, and on top of that, a Cr layer with patterned Ti-based resonators of circular and rectangular multiple patterns. More applications in integrated optoelectronic devices could benefit from the ideal solar absorber's strong absorption, large angular responses, and scalable construction.

## Introduction

All-around complete absorption of omnidirectional and naturally polarized light over a given waveband, which can alternatively be called "blackbody absorbers," is extremely beneficial in solar photovoltaics and other applications such as photodetection and optical modulators^1,2^. Efforts have been made to develop absorbers that are as good as possible. Carbon nanotube forests^3^, silicon nanocones^4^, oxide nanorods^5^, various metallic nanostructures^6^, and so on are some of the common nanomaterials and nanostructures used in blackbody absorbers nowadays. These artificial sub-wavelength structures with controlled optical responses, and metasurfaces, have recently emerged as potential candidates for perfect absorbers. The advantages of metasurface perfect absorbers in managing the light field, including their facile integration, ultra-thin thickness, and high performance, have attracted a lot of research^7,8^. Metal patterns, dielectric spacers, and a metallic layer from top to bottom are the typical sandwich-like configuration of metamaterial perfect solar energy absorbers (MSPSEAs)^9^. It's also worth noting that the originally reported MSPSEAs operate at a single wavelength^10–13^ in the low-frequency range^14^, which limits their practical applicability^15^. As a result, a number of efforts have been made to widen the absorption bandwidth and enhance response frequency. To boost response frequency, the unit cell's feature sizes might be shrunk. Broadening the absorption range can be accomplished in two ways: by overlapping the absorption peaks or by reducing the resonance quality factor^16–20^. Multilayer structure, plasmonic nanocomposites, and gradual size unit cells have all been investigated in order to meet the goals stated above^21^. Another factor that can affect the absorber's performance is the material it's made of. Other materials and dielectrics beyond the more traditional metals and dielectrics have been used to construct wideband MSPSEAs, including TiN, ITO, and even black phosphorus^22–25^. These metasurfaces are expected to attain ultra-wideband (UWB) absorption in recent years and have been demonstrated to have roughly 85 percent absorbance over an ultra-wide operating band, which includes UV to near-infrared (NIR) wavelengths^26^. Because of their difficult production and configuration design, MSPAs are now unable to simultaneously attain ultra-wide working bands and high absorptivity (> 90%). New materials and arrangements are needed to achieve UWB flawless absorption.

Exquisite metals are frequently used in these perfect absorbers due to their plasmon resonance and optical coupling properties^27^. A narrow absorption spectrum is hindered by short storage and expensive costs for the most exquisite metals. In order to keep up with the demand, absorbers with a wider spectrum will be necessary. Using titanium metal, Lui et al. were able to obtain broad absorption across the entire wavelength range^28^. Even at room temperature, titanium is remarkably stable. This refractory metal has a melting point of 1668 degrees Fahrenheit. Additionally, titanium (Ti) metamaterials have been shown to have broadband absorption capabilities^29,30^. Because the imaginary component of the dielectric constant has been greatly reduced, the loss of light absorption can occur over a large frequency range. Plasmonics is regarded to be a major feature of titanium and its composites because of this^31–33^. The plasmon absorption qualities of titanium are due to these characteristics. On the other hand, refractory metals, which can withstand higher temperatures, are more suited for solar absorber materials. Although it is not as abundant as gold, silver, or copper, Titanium is able to successfully address the concerns of low reserves and high costs because its worldwide deposits are significantly bigger. Because of the unique qualities of these refractory metal-based resonant systems, new equipment like solar cells and heat transfer systems can be developed as well as more established ones^34–38^.

In this paper, we have developed an ultrawideband solar energy absorber (UWBSEA) with a high absorption characteristic in the solar spectrum that covers the UV to NIR regions. Due to the metallic layer of titanium nitride, the electromagnetic waves cannot escape the unit cell and contributing to the high absorption and this characteristic is also dependent on the wavelength as well as the thickness of the particular layer. The average absorption response of higher than 90% is observed for the wavelength range of 2670 nm and for the 2000 nm of wavelength range we have achieved more than 95% average absorption. The designing process of the developed UWBSEA is discussed in “Design and modeling” with its results being presented and detailed in “Results and discussion” while “Conclusion” summarizes the paper.

## Design and modeling

**Figure 1:**
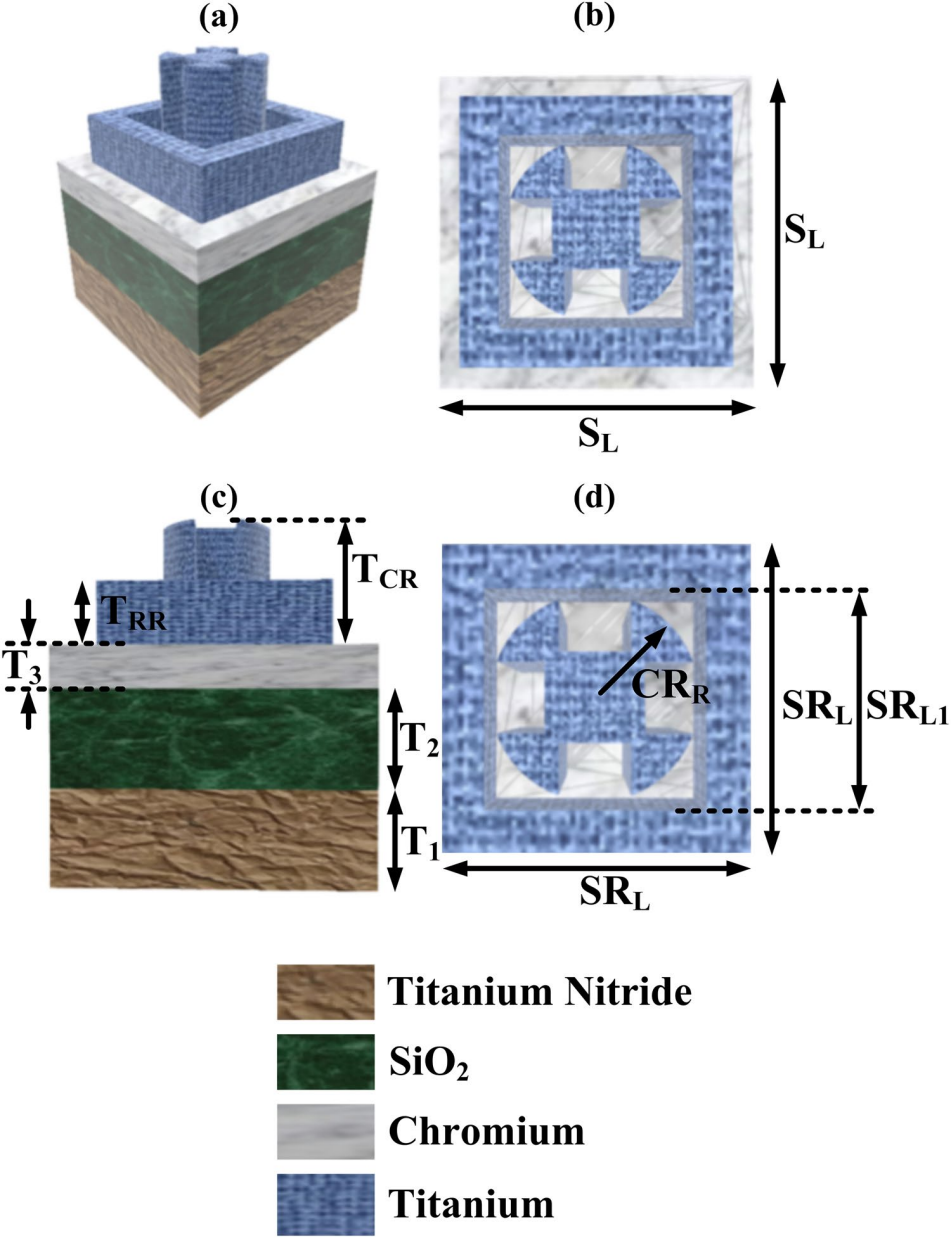
Developed UWBSEA structure (**a**) the structural view of the UWBSEA, (**b**) top view demonstrating the multiple resonator structures of the UWBSEA, (**c**) front view depicting the various layers of the UWBSEA, (**d**) enhanced view of the multiple resonator structures of the UWBSEA and the materials utilized for the construction of the developed UWBSEA.

The UWBSEA is comprised of four layers starting at the bottom layer we have a Titanium Nitride layer, and on that ground layer the second layer of silicon dioxide is placed as we can observe in Fig. 1a. The third layer is of chromium material and it is selected due to its low cost and reflection properties^26^. This structure is the main reason behind achieving the UWB response and the major components contributing to the absorption of the electromagnetic waves. The topmost layer of the rectangular resonator (RR) and circular resonator (CR) is made up of titanium material and thus making the overall resonator structure symmetrical and response contributing to the polarization-independent response. The physical parameters of the proposed UWBSEA are demonstrated throughout Fig. 1b–d with several view representations of the structure including the top and front views. As indicated in Fig. 1b–d, the length of the structure, S_L_, is 500 nm, and the outer and inner lengths of RR, SR_L,_ and SR_L1_, are 400 nm and 300 nm, respectively. The radius of CR, CR_R_, is set at 100 nm. The thickness of the Titanium Nitride layer, T_1_, is fixed at 600 nm, T_2_, and the thickness of the SiO_2_ layer is also set at 700 nm. While the Chromium layer thickness, T_3_ is fixed at 300 nm and the RR thickness, T_RR_, is set at 600 nm and the CR thickness, T_CR_, is decided to keep at 900 nm. The UWBSEA is comprised of three layers designed with TiN, SiO_2_, and Cr materials and a multi resonator structure with Ti material. The FEM approach was used to simulate the UWBSEA's reflection, transmission, and electric field profiles^39^. In all directions, a tetrahedral mesh of 5 nm is employed. With periodic boundary conditions along both x and y, the 3D model of a unit cell is constructed. The z direction was used to5 launch plane waves incident on the unit cell. Titanium (Ti), Titanium Nitride (TiN), SiO_2_, and Chromium material’s complex refractive indices are derived from^40–42^, respectively.

## Results and discussion

**Figure 2:**
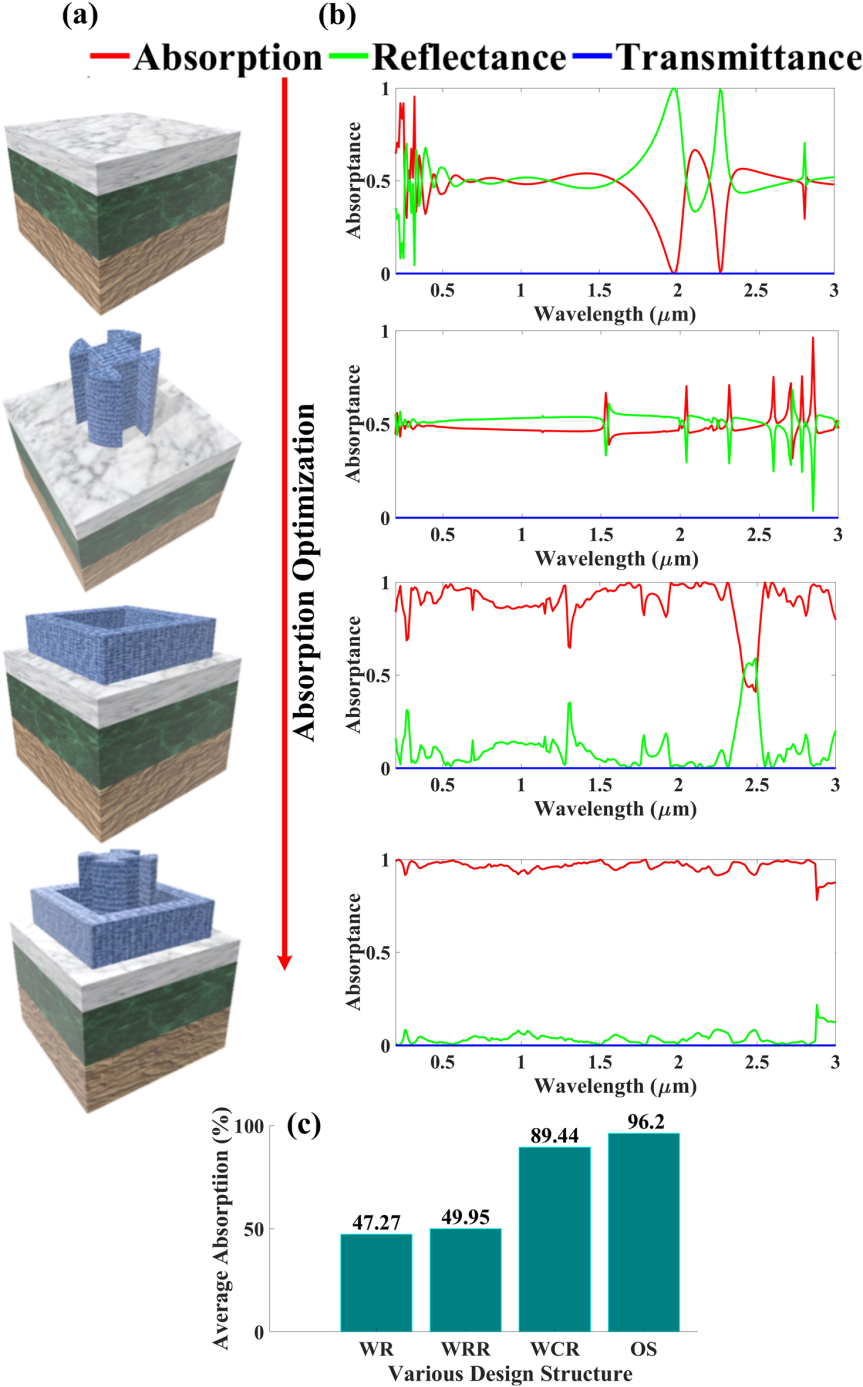
Representation of the step-by-step attainment of the optimized UWBSEA structure, (**a**) multiple variations of SEA structures until the UWB absorption is achieved and the (**b**) corresponding absorption plot, (**c**) bar plot depicting the effect of inserting new structures on the overall average absorption.

**Figure 3:**
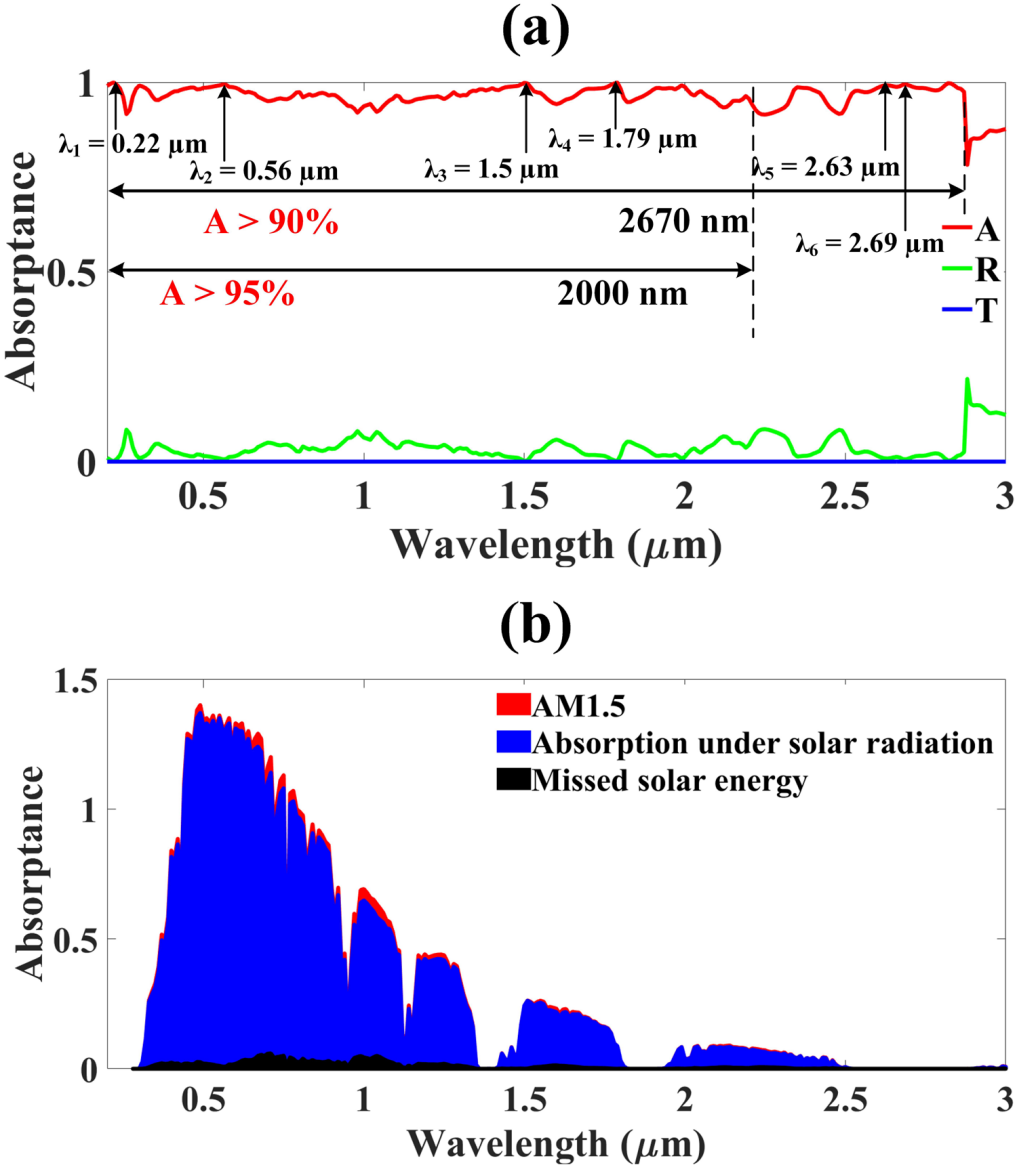
absorption response of the developed UWBSEA (**a**), absorption, reflectance, and transmittance response of the developed UWBSEA, average absorption above 90% is attained for the UWB range of 2670 nm and the average absorption above 95% is observed for the UWB range of 2000 nm with the six near perfect absorption peaks, (**b**) absorption under solar AM1.5 conditions. A very small amount of solar energy is being missed by the developed UWBSEA in the visible range otherwise almost identical absorption is observed.

The FEM method was used to simulate the absorption spectra of various structures, and the simulated results of various structures are shown in Fig. 2. As here, due to the optimal structure that we designed, the achieved transmittance is zero for each and every structure so we can determine absorptivity using A = 1 – R, as T = 0, where R denotes reflection and T is transmittance and this is validated using simulation results presented in Figs. 2 and 3. An illustration of the UWBSEA’s absorption optimization procedure is shown in Fig. 2a. Figure 2b shows the simulated spectra as the absorption performance improves gradually. To begin with, we have employed the three layer structure consisting of the TiN, SiO_2_, and Cr layer without resonator (WR) structures considering the optimization, and this structure provided the overall average absorptance of 47.27% average absorption from UV to mid infrared (MIR) range being 57.27%, 48.3%, 45.03%, 50.41%, respectively and the maximum absorptivity of 95.49% is observed.

In the next structure that we simulated we added the circular resonator of Ti material thickness being 900 nm to the previous structure without a rectangular resonator (WRR) and as we can observe in Fig. 2b, the achieved absorptance response is very fluctuating providing the average overall absorption of 49.95% as shown in Fig. 2c and the absorption in UV to MIR being 49.64%, 48.45%, 50.39%, 49.35%, respectively and for this structure, the maximum absorptivity of 99.56% is attained. Though the second structure attained the near perfect absorptance response in terms of UWB it still lacks and the improvement compared to a previous structure is very negligible that being a mere 2%. The third structure we employed was without a circulator resonator (WCR) and replaced it with a rectangular resonator of the same material with the thickness being 600 nm as indicated in Fig. 2a. The absorption response of this structure observed an average overall absorption of 89.44%, and the average absorption in UV to MIR is 85.67%, 93.42%, 89.69%, and 88.41%, respectively and the observed maximum peak is 99.97%. Here we have observed a drastic change in absorption response and we are also achieving the near perfect absorption peak but if we observe in Fig. 2b around 1100 nm we have a sharp drop in absorption and still, the absorption response is not smooth and achieving spikes. So, to further improve the absorption response and obtain the optimized structure (OS) we employed the structure with the combination of circular as well as a rectangular resonator as shown in Fig. 1a and the absorption results speak for themselves as we can observe the overall average absorption of 96.2% and at the same time, the average absorption in UV to MIR is of 97.04%, 97.90%, 96.12%, and 95.04%, respectively. The near perfect maximum absorptivity of 99.94% is also been observed. As we can observe for each and every band, we are achieving a higher than 95% average absorption and we can conclude that the Ti material layer of CR and RR highly contributes towards achieving the UWB as well as near perfect absorptance response. In Fig. 2 we can observe that for each and every structure we achieve zero transmittance due to the optimal design.

The absorption, transmittance, and reflectance spectra of the developed UWBSEA are demonstrated in Fig. 3a, and we can observe that we achieved the UWB as well as near perfect absorption response. As we can observe in Fig. 3a, from 200 to 2870 nm we are achieving the average absorption response of 90% or more than 90% this being the bandwidth of 2670 nm and in this wavelength range, the average absorption of 96.71% is observed. Other than the proposed UWBSEA also demonstrates the 95% or higher than 95% average absorption response from 200 to 2200 nm (bandwidth of 2000 nm) and in this range, the average absorption of 97.20% is achieved. Apart from this in the simulated range of 200 nm to 3000 nm, the proposed UWBSEA demonstrates the six near unity peaks spread all over from UV to Mir ranges.

As demonstrated in Fig. 3a, the six peaks λ_1_ to λ_6_ are located at 0.22 µm, 0.56 µm, 1.5 µm, 1.79 µm, 2.63 µm, and 2.69 µm with the peak absorptivity of 99.94%, 99.38%, 99.83%, 99.89%, 99.19%, and 99.37%, respectively. As a result, we can say with certainty that the UWBSEA structure has a wide absorption spectrum. Aside from that, the UWBSEA that was developed is capable of absorbing solar energy over a broad wavelength range, from UV to MIR. And as discussed earlier this optimized structure provides the average absorption of 97.04%, 97.90%, 96.12%, and 95.04% from UV to MIR bands as well. The absorption performance of a solar absorber can be determined using a solar spectrogram, an important index. It is possible to get the worldwide spectral equation for AM1.5's incident solar energy using this formula^43^:

1 $${\eta }_{A}=\frac{{\int }_{{\lambda }_{min}}^{{\lambda }_{max}}A\left(\omega \right).{I}_{AM1.5}\left(\omega \right).d\omega }{{\int }_{{\lambda }_{min}}^{{\lambda }_{max}}{I}_{AM1.5}\left(\omega \right).d\omega }$$

The spectrum absorption of a solar absorber at 280–3000 nm can be calculated from Eq. (1) and the solar energy absorbed and missed by the developed UWBSEA is demonstrated in Fig. 3b. In this study, we estimate the amount of solar energy absorbed and lost by installing solar absorbers in the global AM1.5 solar spectrum. In the visible, NIR, and MIR bands of the solar spectrum, the absorbed energy is determined to be over 97.9 percent, above 96.1 percent, and over 95%, respectively, due to the superior absorption performance of the developed UWBSEA in each and every band. As the UWBSEA comes to fruition, it will pave the path for the development of low-cost, high-performance absorbers featuring integrated optoelectronics.

**Figure 4:**
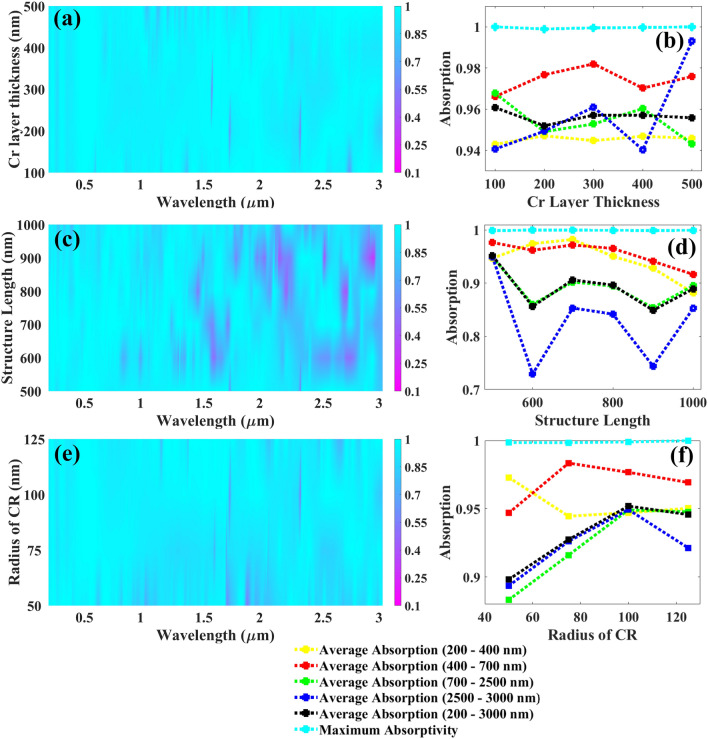
Various UWBSEA physical parameters affecting the absorption response, (**a**) thickness of chromium layer affecting the absorption response, (**b**) line plot indicating the change in average absorption for various regions including UV to MIR as well as maximum absorptivity as we increase the chromium layer thickness from 100 to 500 nm with the gradual change of 100 nm, (**c**) length of a structure affecting the absorption response, (**d**) line plot indicating the change in average absorption for various regions including UV to MIR as well as maximum absorptivity as we increase the structure length from 500 to 1000 nm with the gradual change of 100 nm, (**e**) radius of circular resonator affecting the absorption response, (**f**) line plot indicating the change in average absorption for various regions including UV to MIR as well as maximum absorptivity as we increase the circular resonator radius from 50 to 125 nm with the gradual change of 50 nm.

**Figure 5:**
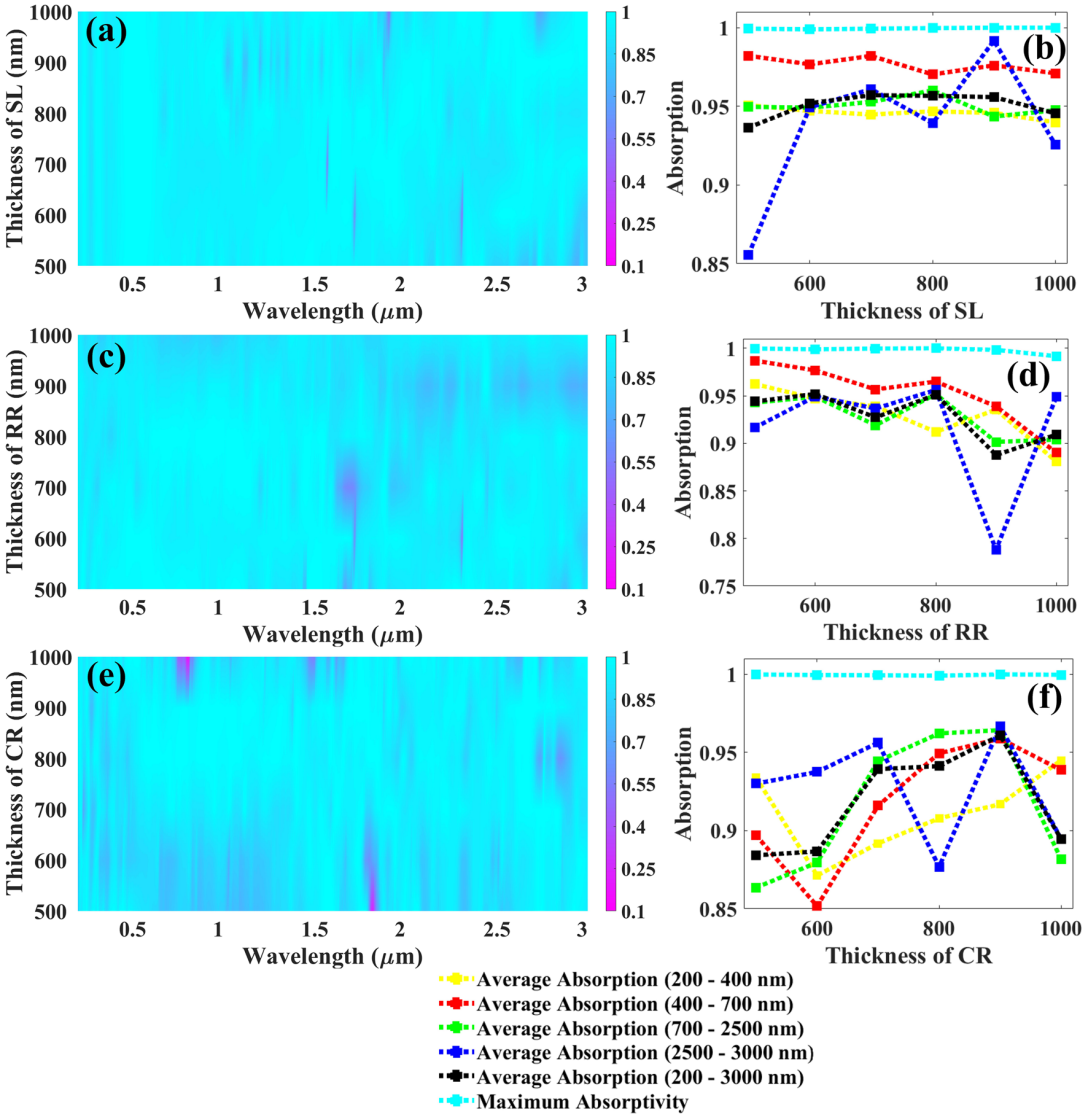
Various UWBSEA physical parameters affecting the absorption response, (**a**) thickness of substrate layer affecting the absorption response, (**b**) line plot indicating the change in average absorption for various regions including UV to MIR as well as maximum absorptivity as we increase the substrate layer thickness from 500 to 1000 nm with the gradual change of 100 nm, (**c**) rectangular resonator thickness affecting the absorption response, (**d**) line plot indicating the change in average absorption for various regions including UV to MIR as well as maximum absorptivity as we increase the rectangular resonator thickness from 500 to 1000 nm with the gradual change of 100 nm, (**e**) thickness of circular resonator affecting the absorption response, (**f**) line plot indicating the change in average absorption for various regions including UV to MIR as well as maximum absorptivity as we increase the circular resonator thickness from 500 to 1000 nm with the gradual change of 100 nm.

The parameter analysis carried out in Figs. 4 and 5, demonstrates that the physical parameters affect the absorption response, as well. Figure 4a demonstrates the color plot indicating the influence of Cr layer thickness increasing from 100 nm with a uniform rise of 100 nm till 500 nm that for all values we are achieving the absorption response above 90% and one cannot decide the best parameter from this figure hence the detailed analysis in terms of absorption is carried out and is depicted in Fig. 4b and as shown in this plot, the average absorption in all the bands are above 94% is observed but at 300 nm we are achieving the average absorptances of 94.48%, 98.20%, 95.28%, and 96.1% from UV to MIR range with the average overall absorption and maximum absorptivity of 95.71%, 99.95%, respectively being the highest for visible, MIR, and overall absorptances and due to this the Cr layer thickness is kept at 300 nm. The impact of structure length varying from 500 to 1000 nm with a uniform rise of 100 nm on the absorption spectrum is depicted in Fig. 4c and we can observe that the absorption is quite identical till 1500 nm for all length values and major variations can be seen after this wavelength and from the detailed absorption analysis shown in Fig. 4d, for 500 nm of structure length we are achieving average absorptances in UV to MIR of 94.7%, 97.67%, 94.91%, and 94.94% are achieved with the maximum absorptivity and the overall average absorption of 99.89%, and 95.19%, respectively. Figure 4e demonstrates the color plot investigating the influence of the radius of CR increasing from 50 nm with a uniform rise of 25 nm to 125 nm for all values we are achieving the almost equivalent absorption response and it is tough to decide the best parameter from this hence the detailed analysis in terms of absorption is carried out and is depicted in Fig. 4f and as shown in this plot, the average absorption in all the bands are increasing till 100 nm and at this value, we are achieving the average absorptances of 94.71%, 97.67%, 94.91%, and 94.94% from UV to MIR range with the average overall absorption and maximum absorptivity of 95.19%, 99.92%, respectively and due to this the CR radius is kept at 100 nm.

Figure 5 demonstrates the impact of various structural thicknesses on the absorption spectrum. The influence of substrate layer thickness varying from 500 to 1000 nm with a uniform rise of 100 nm on the absorption spectrum is depicted in Fig. 5a and we can observe that the absorption is quite identical for all values and one cannot decide the best parameter from this figure hence the detailed absorption analysis shown in Fig. 5b, for 700 nm of structure length we are achieving average absorptances in UV to MIR of 94.48%, 98.2%, 95.28%, and 96.1% are achieved with the maximum absorptivity and the overall average absorption of 99.93%, and 95.71%, respectively and so this thickness is kept at 700 nm. Figure 5c demonstrates the color plot indicating the influence of RR thickness increasing from 500 nm with a uniform rise of 100 nm till 1000 nm that for all values we are achieving the absorption response above 90% and from the detailed absorption analysis carried out in Fig. 5d, the average absorption in all the bands is above 90% for most of the thicknesses is observed and at 500 nm itself we are achieving the highest average absorptances of 96.22%, 98.70%, 94.28%, and 91.67% from UV to MIR range with the average overall absorption and maximum absorptivity of 94.4%, 99.97%, respectively. The impact of the thickness of CR varying from 500 to 1000 nm with a uniform rise of 100 nm on the absorption spectrum is depicted in Fig. 5e and we can observe that the absorption is getting affected a lot and major variations can be seen and to obtain a proper understanding we have carried out the detailed absorption analysis depicted in Fig. 5 (f), for 900 nm of CR thickness we are achieving highest average absorptances in UV to MIR of 91.68%, 95.88%, 96.942%, and 96.968% are achieved with the maximum absorptivity and the overall average absorption of 99.99%, and 96.09%, respectively.

**Figure 6:**
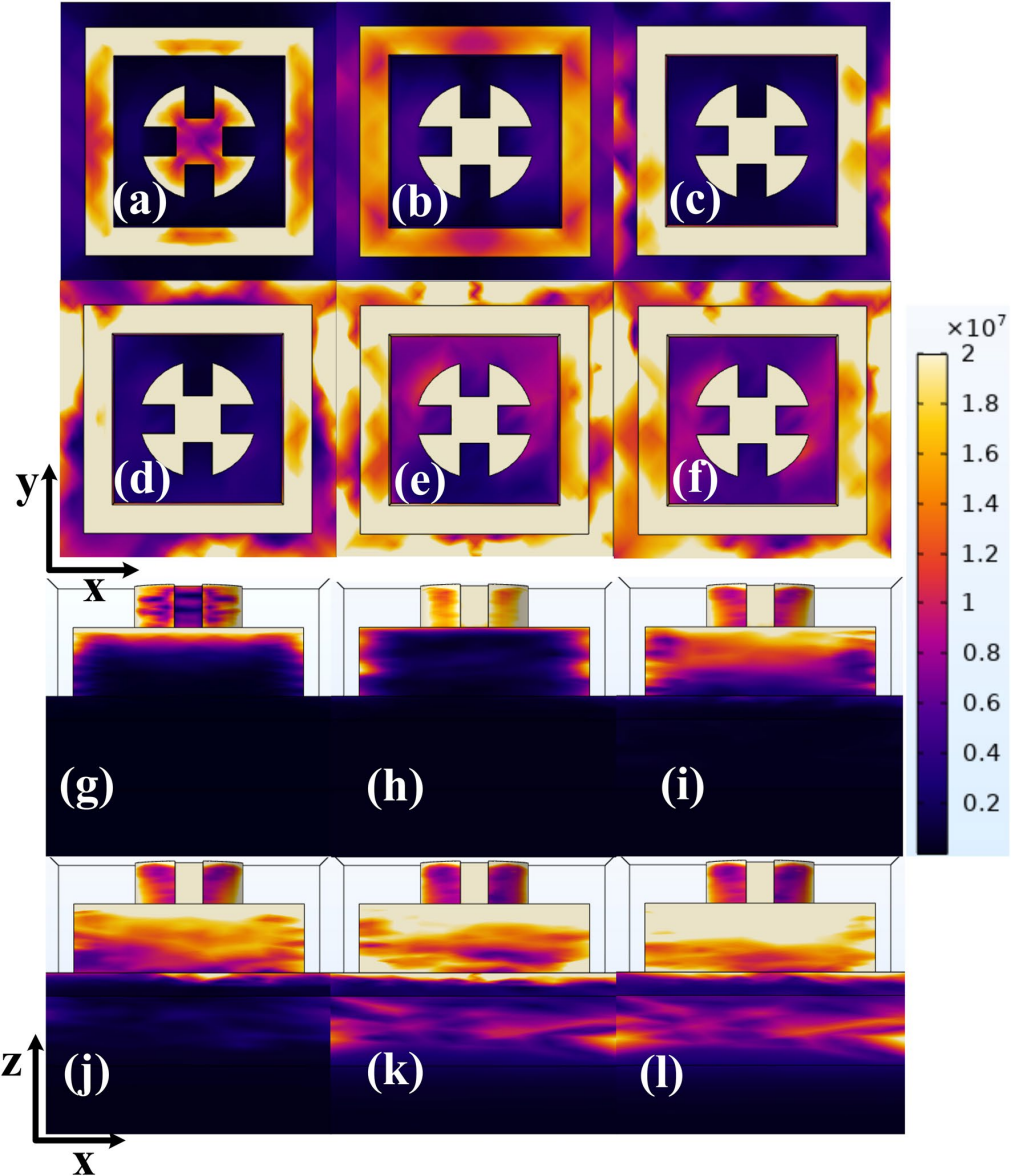
Representation of Electric field distribution of the developed UWBSEA at the six near perfect absorption peaks with two different views i.e., XY and XZ, respectively (**a,g**) 0.22 µm, (**b,h**) 0.56 µm, (**c,i**) 1.5 µm, (**d,j**) 1.79 µm, (**e,k**) 2.63 µm, (**f,l**) 2.69 µm.

Furthermore, in order to explain how the developed UWBSEA can generate broadband absorption at 0.22 µm, 0.56 µm, 1.5 µm, 1.79 µm, 2.63 µm, and 2.69 µm, electromagnetic field distributions (EFD) are simulated and demonstrated for two views including XY and XZ with their depiction in Fig. 6a–f and g–l, respectively. The first peak of 99.94% at 220 nm is observed and the maximum EF is only spread over the rectangular and minor EF is spread over the circular resonator structures as depicted in Fig. 6a,g. At 560 nm the second peak of 99.38% is observed and in the EFD plot from Fig. 6b,h a minor amount of EF is found across the rectangular resonator while most of the EF is concentrated over the circular resonator.

The third peak of 99.83% at 1500 nm is observed and the maximum EF is spread all over the rectangular and circular resonator structures with a deeper EF found at the lower part of the rectangular resonator compared to previous cases as depicted in Fig. 6c,i. At 1790 nm the fourth peak of 99.83% is observed and in the EFD plot from Fig. 6d,j a higher amount of EF is found across the rectangular resonator and the circular resonator as well here we also got the maximum EF concentration on the outer surface of CR as well as RR surface. Here we can also observe the small amount of EF over the Chromium and SiO_2_ layer. The fifth peak of 99.19% at 2630 nm is observed and the same amount of EF is spread all over the rectangular and the circular resonator structures as observed in previous cases with a higher EF concentration found at Cr and SiO_2_ layers compared to previous cases as depicted in Fig. 6e,k. At 2690 nm the sixth peak of 99.37% is observed and in the EFD plot from Fig. 6f,l a higher amount of EF is found across the rectangular resonator and the circular resonator as well here we also got the maximum EF concentration on the outer surface of CR as well as RR surface with the maximum EF concentration spread across the Cr and SIO_2_ layers. The one thing common across these structures is that the ground layer of TiN contributes towards the transmittance extinction hence no EF concentration is found and as a result zero absorption in this layer.

**Figure 7:**
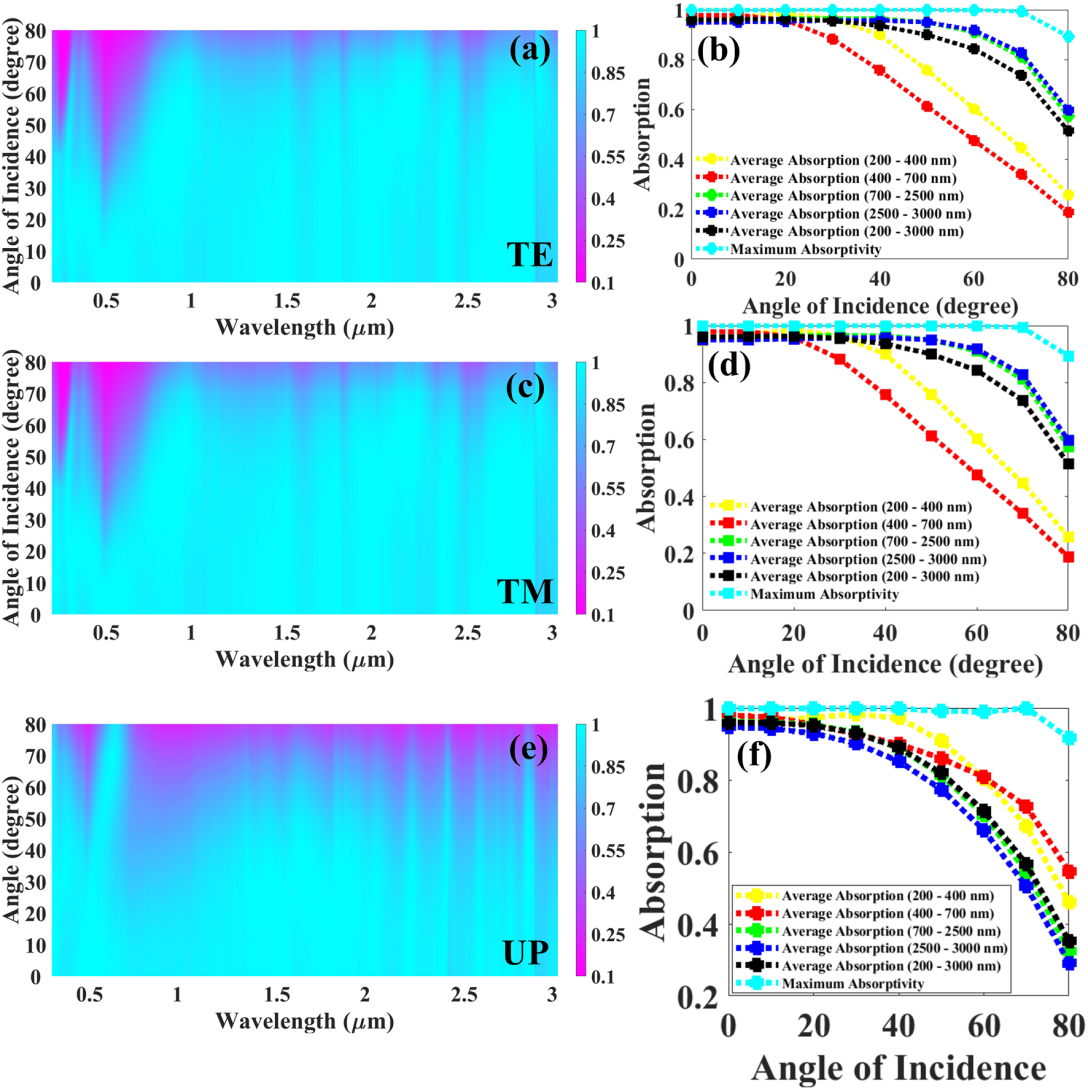
UWBSEA’s absorption response for various IA, (**a**) absorption response for IA for TE, (**b**) line plot indicating the change in average absorption for various regions including UV to MIR as well as maximum absorptivity as we increase the substrate layer thickness from 0 to 80 degree with the gradual change of 10°, (**c**) absorption response for IA for TM, (**d**) line plot indicating the change in average absorption for various regions including UV to MIR as well as maximum absorptivity as we increase the IA from 0° to 80° with the gradual change of 10°, (**e**) absorption response for IA for unpolarized light, (**f**) line plot indicating the change in average absorption for various regions including UV to MIR as well as maximum absorptivity as we increase the IA from 0° to 80° with the gradual change of 10°.

The solar absorber must be polarization independent and insensitive to large incident angles (IA) in order to be widely employed in nature. Absorptivity is plotted against incidence angle and wavelength in Fig. 7 for several light sources with various polarization states. Figure 7a depicts the absorption response under TE polarized light and from the figure, it is visible that we start to observe the drop in absorption in visible and UV bands after 30 degrees and after further detailed analysis in terms of absorption as demonstrated in Fig. 7b, the overall average absorption, average absorption in NIR MIR is still above 90% for the IA of 60 degrees and we achieve the highest average absorptances of 60.36%, 47.6%, 91%, and 91.81% in UV to MIR bands. The gradual drop in average absorptance of UV and visible bands is observed after 30 degrees IA. The absorption response affected by TM polarized light is shown in Fig. 7c and we can say from both Fig. 7c,d that the TE and TM polarized lights affect the developed UWBSEA identically proving the polarization-independent characteristics of the proposed UWBSEA. Figure 7e depicts the absorption response under unpolarized (UP) light and from the figure, it is visible that we start to observe the drop in absorption in every band after 50 degrees and after further detailed analysis in terms of absorption as demonstrated in Fig. 7f, the overall average absorption is still above 80% for the IA of 50 degrees and we achieve the highest average absorptances of 90.9%, 86%, 81.37%, and 77.4% in UV to MIR bands. The gradual drop in average absorptance of UV and visible bands is observed after 70 degrees IA. So, here we can clearly state that the proposed structure can absorb highly under unpolarized light conditions compared to the TE and TM conditions. Furthermore, for unpolarized light, we achieve higher absorption in the visible region for higher IA and as the concentration of solar radiation is high in this range these results clearly embark on a potential application for the improvement of photovoltaic devices as well. The presented structure’s resonator structure is symmetrical and hence we have achieved the identical absorption response for both TE and TM modes. Our structure’s absorption response varies slightly in a very small range only for the angle of incidence till 60° for the visible region as till 60° we achieve the higher absorption for each region except visible and it is further degraded for the rest of the angles. To resolve this issue, we can construct a platform to avoid the ray’s incidence at these particular angles and achieve the proper absorption response to improve the performance of photovoltaic devices.

It's clear from Table 1 that our UWBSEA has significant benefits over other absorbers. We would like to point out that each and every material used here from Ti to SiO_2_ is easily available and cost-effective materials compared to the precious materials that are generally used for the solar absorber. Furthermore, the thickness of each and every material used for the proposed structure is below 1000 nm. The first three layers, i.e., TiN, SiO_2_, and Cr can be easily deposited with the help of thin film deposition, and later the Ti based resonator is simply a rectangle and cylinder based structure that can be easily fabricated with the help of lithography as the presented structures are very basic. So, we can state that the proposed structure is simpler and faster to construct. Secondly, our absorber's absorption efficiency is 95 percent for 2000 nm and 90 percent for 2670 nm which is higher than other absorbers, making it superior. According to our results, our absorber has an excellent average absorption efficiency when compared to a weighted average absorption efficiency at AM1.5. When everything is taken into account, it's clear that solar absorbers are an important part of solar absorption because of their simple design and high performance.

**Table 1:** Comparison study of developed UWBSEA with available literature.

Design	Range	% Average absorption overall	Bandwidth (absorption above 90%) (nm)	Bandwidth (absorption above 95%) (nm)	Polarization insensitive
**Developed UWBSEA**	**200–3000 nm**	**96.2**	**2670**	**2000**	**Yes**
Ref^44^	0.2–3 µm	94.42%	2680	1310	**Yes**
Ref^21^	0.1–3 µm	95	2516	–	Yes
Ref^45^	0.1–2 µm	93.17	1759	–	Yes
Ref^23^	0.3–3 µm	Higher than 90	2196	–	Yes
Ref^25^	0.3–1.624 µm	95	1264	–	–
Ref^46^	0.4–2 µm	Higher than 90	1110	–	Yes
Ref^30^	0.4–2 µm	Higher than 90	1007	–	Yes
Ref^47^	295–2500 nm	93.26	1650	–	Yes
Ref^48^	300–2500 nm	Higher than 90	1310	–	Yes

Significant values are in bold.

## Conclusion

Several materials and designs have been attempted in quest of the ultimate ultra-broadband light absorber, achieving ultra-broadband and strong unpolarized light absorption over a broad angular range has proven to be a significant challenge. Optical metamaterials' light-field regulation capabilities are a potential new solution for perfect absorbers. Our objective is to create and demonstrate an ultra-broadband solar absorber for the ultraviolet to the mid-infrared range with an average TE/TM light absorption of 96.2%. In the visible, NIR, and MIR regions of the solar spectrum, according to the AM1.5 spectrum analysis, the absorbed energy is greater than 97.9%, above 96.1%, and greater than 95%, respectively. Apart from this for the 2000 nm of wavelength range, we achieved more than 95% average absorption in which the average absorption is 97.2% and for the 2670 nm wavelength range the average absorption of more than 90% is achieved with an average absorption of 96.71%. In order to produce this wideband absorption, the TiN material ground layer is followed by the SiO_2_ layer, and then a Cr layer with many circular and rectangularly designed Ti-based resonators. More integrated optoelectronic device applications could profit from the perfect solar absorber's high absorption, large angular responses, and scalability.

## Data Availability

Data will be made available at a reasonable request to corresponding author.
